# Voxel level quantification of [^11^C]CURB, a radioligand for Fatty Acid Amide Hydrolase, using high resolution positron emission tomography

**DOI:** 10.1371/journal.pone.0192410

**Published:** 2018-02-14

**Authors:** Pablo M. Rusjan, Dunja Knezevic, Isabelle Boileau, Junchao Tong, Romina Mizrahi, Alan A. Wilson, Sylvain Houle

**Affiliations:** 1 Research Imaging Centre, CAMH Campbell Family Mental Health Research Institute, Toronto, Ontario, Canada; 2 Department of Psychiatry, University of Toronto, Toronto, Ontario, Canada; Wayne State University, UNITED STATES

## Abstract

[^11^C]CURB is a novel irreversible radioligand for imaging fatty acid amide hydrolase in the human brain. In the present work, we validate an algorithm for generating parametric map images of [^11^C]CURB acquired with a high resolution research tomograph (HRRT) scanner. This algorithm applies the basis function method on an irreversible two-tissue compartment model (*k*_4_ = 0) with arterial input function, i.e., BAFPIC. Monte Carlo simulations are employed to assess bias and variability of the binding macroparameters (*K*_*i*_ and λ*k*_3_) as a function of the voxel noise level and the range of basis functions. The results show that for a [^11^C]CURB time activity curve with noise levels corresponding to a voxel of an image acquired with the HRRT and reconstructed with the filtered back projection algorithm, the implementation of BAFPIC requires the use of a constant vascular fraction of tissue (5%) and a cutoff for slow frequencies (0.06 min^-1^). With these settings, BAFPIC maintains the probabilistic distributions of the binding macroparameters with approximately Gaussian shape and minimizes the bias and variability for large physiological ranges of the rate constants of [^11^C]CURB. BAFPIC reduces the variability of *K*_*i*_ to a third of that given by Patlak plot, the standard graphical method for irreversible radioligands. Application to real data demonstrated an excellent correlation between region of interest and BAFPIC parametric data and agreed with the simulations results. Therefore, BAFPIC with a constant vascular fraction can be used to generate parametric maps of [^11^C]CURB images acquired with an HRRT provided that the limits of the basis functions are carefully selected.

## Introduction

Fatty acid amide hydrolase (FAAH, EC3.5.1.99) is the major metabolizing enzyme responsible for terminating the action of the endocannabinoid anandamide (N-arachidonoylethanolamide, AEA) and other fatty acid amides (e.g., oleoylethanolamide (OEA) and palmitoylethanolamide (PEA)). As such, FAAH sets the tone for endocannabinoid signaling and therefore modulates what is believed to be a range of human behaviors and processes including motor, pain, inflammation, pregnancy, appetite, mood, cognition, and addiction. We recently developed and evaluated [^11^C-carbonyl]URB694 ([^11^C]CURB) for positron emission tomography (PET) quantification of FAAH binding in the human brain[1, 2]. Using a region of interest (ROI) analysis and an arterial plasma input function, we found that the irreversible 2-tissue compartment model (2-TCMi) provided an accurate fitting of the time activity curves (TAC). Additionally, the net influx constant *K*_*i*_ and the composite parameter λ*k*_3_ with λ = *V*_ND_ = *K*_1_/*k*_2_ had a coefficient of variation (*CoV*) less than 5% with 60 minutes of scan data and the uptake of [^11^C]CURB was far from being flow-limited (*k*_3_/*k*_2_~0.55) [3]. Using PF-04457845[4, 5], a highly specific FAAH inhibitor, we confirmed that the first compartment of the 2-TCMi represents free and non-specific binding[2].

While the ROI level analysis is the preferred method when the anatomical localization of the areas of interest can be hypothesized, the voxel level analysis maximizes the potential of exploratory and/or data driven analyses. However, voxel level quantification is challenging as a result of the inherent noisy data. Due to the computational demands and numerical deficiencies to converge to the global minimum, non linear fittings of compartmental models are not usually a viable option for the creation of parametric maps (i.e. images containing the kinetic parameter value for each voxel) and as a result several approaches have been developed [6]. The classical methods are based on linearization of the kinetic equations (i.e. graphical analysis), which strongly reduces the computational demands. The most widely used method for irreversible radioligands with an input function is the Patlak plot[7]. This method does not assume a number of reversible compartment; however, it requires that the slowest reversible compartment is in effective equilibrium with the tracer in plasma[8], otherwise the estimation of the net uptake constant (*K*_i_) will be biased. At the high noise level of a small voxel’s TAC, the Patlak plot produces unacceptable variability for the images studied here (see results), demonstrating the need for an alternative method. Basis function methods (BFM)[9–11] allow for the linearization of the kinetic model equations using a family of basis functions. BFMs are known for reducing the variability of the parameter estimations. The application of BFMs, for an irreversible two-tissue compartment model with arterial input function, was recently introduced as BAFPIC[12]. While the outcome of Patlak plot is limited to *K*_i_, BAFPIC can produce estimations of the individual rate constants. The members of the basis function family are the convolution of monoexpontial functions of different decay time constants (sometimes referred to as “frequencies”) with the arterial input function. BFMs can be seen as a specific case of spectral analysis[13] in which the number of monoexponential convolutions to model a radioligand is known (e.g. radioligands, whose kinetics are described by the 2-TCMi, will activate only a single frequency of the frequency spectrum when analyzed with spectral analysis[14]). .Low frequency exponentials in spectral methods are known to produce biases in the estimation of the parameters[15]. This bias, however, has not been studied for BAFPIC, in which the frequencies (θ) have a simple expression as function of the rate constants (*θ* = *k*_2_*+k*_3_).

The goal of the present work is to validate the generation of parametric maps of [^11^C]CURB using BAFPIC with images acquired on a high resolution research tomograph (HRRT, CPS/Siemens, Knoxville, TN, USA). The small crystal dimension in the HRRT tomograph results in improved spatial resolution, though with a concomitant reduction in the reconstructed voxel signal to noise. This reduction in voxel signal to noise is the direct consequence of the crystal dimension which defines the sinogram sampling distance. A smaller voxel size, additionally, allows a more precise description of the anatomical structure. Hence it is both, an excellent and challenging tool to investigate radioligand binding at the voxel level.

In this work, we first determined the optimal setting for BAFPIC using simulations and later, using experimental human data, we validated the predictions of the model. The shape of the probability distribution, bias and variability of *K*_i_, *λk*_*3*_ and the fractional blood volume (*V*_*B*_) were studied using computer simulations which included: TACs with multiple sets of realistic combinations of the rate constants values, TACs with different noise levels, basis function with different ranges and numbers, and the use of *V*_*B*_ as a constant or as a variable. Results were compared with those from Patlak plot. Finally, the simulated results were confronted with real data. A comparison between region of interest analysis and voxel by voxel analysis was performed using a Bland-Altman plot for 6 healthy subjects at baseline and in the blocked condition using the FAAH inhibitor, PF-04457845. The expected performance of BAFPIC predicted by the simulations are presented as a function of voxel noise level and FAAH concentrations; therefore, the results of the work can immediately be applied to images of [^11^C]CURB obtained with other scanners, algorithms of reconstruction or denoising filters post-reconstruction.

## Material and methods

### Kinetic analysis

Following the definitions proposed in the consensus nomenclature for radioligands[16], the TAC *C*_*T*_(*t*) of a radioligand described by an irreversible 2TCM with metabolites-corrected arterial input function *C*_*a*_(*t*) and radioactivity in the vascularity *C*_*b*_(*t*) can be described as [17]:

$$C_{T}\left(t\right)=\frac{\left(1-V_{B}\right)}{\theta }K_{1}k_{3}\int_{0}^{t}C_{a}\left(\tau \right)d\tau +\frac{\left(1-V_{B}\right)}{\theta }K_{1}k_{2}e^{-\theta t}⊗C_{a}\left(t\right)+V_{B}C_{b}\left(t\right)$$ (1)

Where

$$\theta =k_{2}+k_{3}$$ (2)

Using a family of basis functions (BF_j_, *j = 1*..*n*) of *n* exponential convolution of the input function:

$${\mathrm{BF}}_{j}\left(t\right)=e^{-\theta_{j}t}⊗C_{a}\left(t\right)$$ (3)

with *θ*_*j*_ logarithmically spaced in the range [*θ*_*min*_, *θ*_*max*_][11, 18], Eq 1 can be converted in *n* linear equations of the shape:

$$\left[\begin{array}{c} \phi_{1}\\ \phi_{2}\\ V_{b} \end{array}\right]=A_{j}^{-1}WC_{T}$$ (4)

Where *A_j_* = *W*[∫*C_a_*(*t*) BF*_j_*(*t*) *C_b_*(*t*)], *ϕ*_*1*_ *= (1-V*_*B*_*)K*_*1*_*k*_*3*_*/θ*, *ϕ*_*2*_ *= (1-V*_*B*_*)K*_*1*_*k*_*2*_*/θ* and *W* is a diagonal matrix of weight of the data points. $A_{j}^{-1}$ can be computed using QR decomposition.

The linear equation for the value *θ*_*j*_ that minimizes the weighted residual sum of squares is chosen as the optimal solution. The rate constants can be computed as:

$$K_{1}=\left(\phi_{1}+\phi_{2}\right)/\left(1-V_{B}\right),\;k_{2}=\phi_{2}\theta /\left(\phi_{1}+\phi_{2}\right),\;k_{3}=\phi_{1}\theta /\left(\phi_{1}+\phi_{2}\right)\;\mathrm{and}\;K_{i}=\phi_{1}/\left(1-V_{B}\right)$$

Eventually, *V*_*B*_ can be assumed as a constant rather than a variable, and thus the vascular contribution can be subtracted from the TAC prior to solving the equation. In this case, *A*_*j*_ = *W*[∫*C*_*a*_(*t*) BF_*j*_] and we will merely have two variables (*ϕ*_1_ and *ϕ*_2_). While in a large gray matter region, which is composed of a mixture of capillaries and brain tissue, it is expected that *V*_*B*_~5%, this simplification is conflictive at a voxel level as it can be entirely inside of an artery (*V*_*B*_ = 100%).

*K*_i_ of irreversible radioligands is usually estimated with the Patlak plot[7]. $K_{i}^{Patlak}$ corresponds to the slope of the plot *C*_*T*_(*t*)/*C*_*a*_(*t*) vs ∫*C*_*a*_(*t*)/*C*_*a*_(*t*) after a time t* in which it reaches linearity. It takes place after all the reversible compartments in the system have reached effective equilibrium with the plasma compartment[8]. Patlak plot is usually applied subtracting a vascular contribution with a given $V_{B}^{Patlak}$ from the TAC. Our previous ROI analysis of [^11^C]CURB showed that the Patlak plot underestimates the *K*_i_ value given by the 2TCMi [1]. In the present work, Patlak plot was applied after correcting the TAC for a 5% of vascular contribution.

### Simulations

#### Rate constants for TAC

Monte Carlo simulations were performed to assess 1) the shape of the distribution, bias and variability of *K*_i_, *λk*_*3*_ and *V*_*B*_ as a function of noise and range [*θ*_*min*_, *θ*_*max*_], 2) bias and variability introduced by fixing *V*_*B*_ to an incorrect value. Patlak plot was also performed for comparison.

The main simulations used in this work were based on the 60 minutes decay corrected TAC created by the rate constant of an average “putamen” ($K_{1}^{P}$ = 0.31 mL·cm^-3^·min^-1^, $k_{2}^{P}$ = 0.1 min^-1^ and $k_{3}^{P}$ = 0.049 min^-1^)[1].

Changes in regional cerebral blood flow (rCBF) were simulated by changing *K*_1_. Our previous results^1^ led to a very low rCBF (~20 mL·100 mg^-1^·min^-1^) with *K*_1_ = 0.16 mL·cm^-3^·min^-1^ and a very high rCBF (~110 mL·100 mg^-1^·min^-1^) with *K*_1_ = 0.36 mL·cm^-3^·min^-1^. Changes in *V*_*ND*_ were simulated by changing *k*_*2*_ for a given *K*_1_ value. Previous results showed that *V*_*ND*_ is within the range 2 to 4 mL·cm^-3^. Additional simulations were performed with combination of those maximum and minimum rCBF and *V*_*ND*_ values and with typical rCBF (*K*_1_ = 0.31 mL·cm^-3^·min^-1^) and very high *V*_*ND*_ = 4.5 mL·cm^-3^.

Changes in *B*_max_ (FAAH activity) were modeled by multiplying $k_{3}^{P}$ by 0.1, 0.2, 0.35, 0.5, 0.75, 1, 1.25, 1.5 and 2.

The same arterial unmetabolized radioligand in plasma (input function) and whole blood curve of a typical subject were used for all the simulations. In all simulations, $V_{B}^{TAC}$ = 5% except in the simulation for examining the bias caused by fixing *V*_*B*_ to a wrong value in BAFPIC ($V_{B}^{TAC}$ = 0%, 5%, 10% and 25%).

### Setting for the basis functions (BFs): Range and number

**Fig 1:**
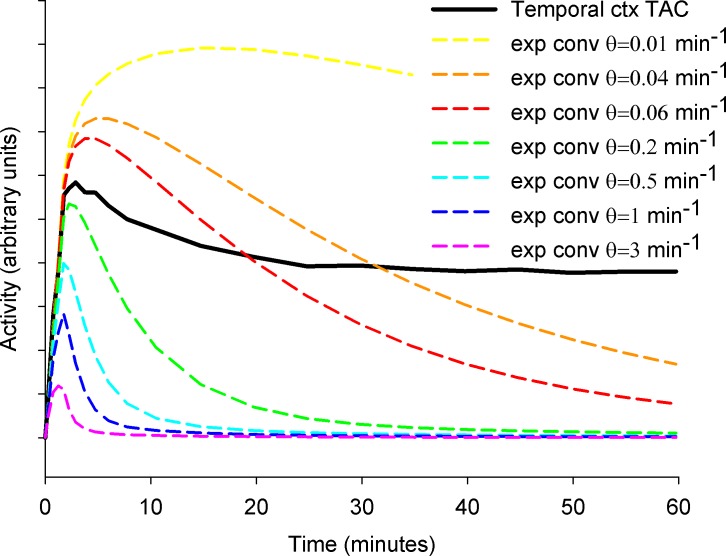
Exponential convolution of the input function of a single subject for different time constant θ compared with a regional TAC (temporal ctx) for the same subject. The TAC’s peakwidth is mostly given by a single basis function. Taking into account different regional TACs, it gives an idea about the set of elements to include in the basis.

Given that *θ = k*_*2*_*+k*_*3*_ and *k*_*3*_ *= k*_*on*_*B*_max_, a BF with ${\theta_{min}<k}_{2}^{min}$ applied to Eq 1 should describe the TAC of a voxel without FAAH (*k*_*3*_ = 0) and a BF with ${\theta_{max}>k}_{2}^{max}+k_{3}^{max}$ should describe the TAC of the maximum expected *B*_max_. While *θ*_min_ = min (*θ*) will produce the BF that washes out slowest, *θ*_max_ = max(*θ*) will produce the quickest BF washing out (Fig 1).

From our ROI based analysis of [^11^C]CURB, we have learned that by accounting for one standard deviation, $k_{2}^{min}$ = 0.07 min^-1^ (caudate), $k_{2}^{max}$ = 0.13 min^-1^ (temporal ctx) and $k_{3}^{max}$ = 0.067 min^-1^ (cerebellum)^1^. Therefore, the BF set explored in this work includes: *θ*_min_ = 0.01, 0.02, 0.03, 0.04, 0.05, 0.06 and 0.07 min^-1^ and *θ*_max_ = 0.2, 0.5, 1 2, 3 min^-1^. We studied BF with *n* = 50, 100 250, 500 and 1000 member logarithmically separated between *θ*_min_ and *θ*_max_.

For clarity, in the rest of the manuscript, the BF will be described as n = 50 *frequencies* logarithmically spaced between [*θ*_min_, *θ*_max_]

### Noise and weight for fittings

Noise for the frame *i* from time $t_{i}^{s}$ to $t_{i}^{e}$ was modeled with a Gaussian distribution with standard deviation (*SD*_*i*_):[19]

$$SD_{i}=sf\sqrt{\frac{e^{\lambda_{c}\left(t_{i}^{e}+t_{i}^{s}\right)/2}C_{i}}{\left(t_{i}^{e}-t_{i}^{s}\right)}}$$ (5)

where *C*_*i*_ is the noise-free simulated radioactivity, *λ*_*c*_ = 0.0339 min^-1^ is the decay constant of ^11^C and *sf* is the scale factor that controls the noise level. The mean percent noise contained in the noisy data was calculated as[20]: %∑_*i*_*SD*_*i*_/∑_*i*_*C*_*i*_. *sf* = 7 is characteristic of noise in a typical sized ROI, *sf* = 20 of a very small ROI (i.e. anterior cingulate cortex) and *sf* = 100~120 of a single voxel in the gray matter of an image acquired and reconstructed as previously published[1, 2] (i.e acquired by an HRRT following a bolus injection with 10 mCi of [^11^C]CURB and reconstruction using the 2D filtered-back projection (FBP) algorithm, with a HANN filter at Nyquist cutoff frequency. For details see [1]). The Coefficient of variation (*CoV*) reported in the simulation was calculated as the standard deviation/mean.

### Human studies

The human analysis presented here is a parametric maps analysis of a study previously published using a region of interest (ROI) approach[2]. The protocol was approved by the Center for Addiction and Mental Health Ethics Review Board and conformed to the Declaration of Helsinki. All subjects provided written informed consent after all study procedures were fully explained. Images of six healthy volunteers (3 men and 3 women; aged 19–53 years) were acquired before and 2 hours after an oral dose of a potent specific FAAH blocker, PF-04457845. A saline solution of 370 ± 40 MBq (10± 1 mCi) of [^11^C]CURB was injected over a 1-minute period at a constant rate using a Harvard infusion pump (Harvard Apparatus, Holliston, MA, USA) into an intravenous line placed in an antecubital vein. The images were reconstructed into 22 time frames. The first frame was of variable length dependent on the time between the start of acquisition and the arrival of [^11^C]CURB in the tomograph field of view (FOV). The subsequent frames were defined as 5x30 sec, 1x45 sec, 2x60 sec, 1x90 sec, 1x120 sec, 1x210 sec and 10x300 sec. All images were decay corrected. The arterial blood analysis, input function creation and delay and dispersion calculation were previously described[1, 2]. Results of the blocking study in the white matter (WM) were not previously explored. WM delineation was performed following the algorithm described in Bencherif et al.[21], which includes WM predominately from the corpus callosum, allowing for a maximum of 5% partial volume effect from the gray matter. Head movement in the dynamic PET acquisition was corrected using a frame-by-frame realignment of images reconstructed iteratively unweighted OSEM (3 iterations, subset 6, span 3) without attenuation correction[22]. TACs were fitted assuming *V*_*B*_ = 5% [23] and data point weighted based on the trues in the field of view[24].

## Results

### Simulations

Fig 1 shows the *BF*_*j*_*(t)* for a typical input function and a set of values *θ*_*j*_. The position of the peak of *BF*_*j*_*(t)* increases when *θ*_*j*_ decreases.

Considering Fig 1 and Eq 1, the position and width of the peak of the TAC of [^11^C]CURB is given mainly by *BF*_*j*_*(t)*. Therefore, comparing the position of the peak of BFs respect to the TAC can help to verify whether the range [*θ*_min,_ *θ*_max_] is reasonable. Typical TACs for [^11^C]CURB show a peak between 120 to 260 seconds after injection[1]. For *θ≥*0.2 min^-1^ the peak, before 180 sec after injection, will be too early for some regional TACs. For *θ≤*0.02 min^-1^ the peak will be too late (after 500 seconds). Thus, *θ* = [0.06, 0.2] min^-1^ represents a conservative initial estimation for the range of *θ* for a TAC of [^11^C]CURB in a gray matter region of a healthy subject.

Results of the simulations are reported in a Microsoft Office Excel file (S1 File).

### Number of simulations

The percentage error of the mean (*E*) in *n*_*c*_ Monte Carlo simulations for a given confidence interval with critical value *z*_*c*_ can be estimated as $E=\frac{100z_{c}S_{x}}{\bar{x}\sqrt{n_{c}}}$, where $\bar{x}$ and *S*_*x*_ are the sample mean and sample standard deviation for a large number of simulations. As it will be seen below, $\bar{x}$ and *S*_*x*_ were highly dependent on the data simulated, the noise level and the model used for quantification. The results presented in this work have been calculated using *n*_*c*_ *= 5000*, which using a confidence level of 95% (*z*_*c*_ = 1.96) led to *E*<<5% in most of the scenarios studied. Exceptions occurred in cases with a very low $\bar{x}$ and a very high *S*_*x*_, which have been observed using BAFPIC with a low θ_min_ (0.01 min^-1^) or Patlak model on data with high levels of noise (sf = 120) and low specific binding ($k_{3}/k_{3}^{P}$ = 0.1 or 0.2).

### Simulation results using BAFPIC with constant VB = 5%

#### Minimal basis set

Basis set including *n* = 2,3,4,5,10,25,50,100,250,500 and 5000 members were evaluated in the largest range of *θ* = [0.01, 3] min^-1^ studied and the optimal range of *θ* = [0.06, 3] min^-1^ (see below). At any noise level *sf*>7, n>50 does not produce further evolution in the probability distribution of *K*_i_ and *λk*_3_ (regarding mean, standard deviation (*std*), kurtosis (*kurt*) and skewness (*skew*)). Results presented in this manuscript were calculated using *n* = 50.

Interestingly, when the noise level decreases, more BFs are required. At *sf* = 7, a noise level of a middle sized ROI TAC (i.e putamen), *n* = 100 was required when the largest range [0.01, 3] min^-1^ was used. The discretization of *θ* becomes more inefficient for TACs with lower noise: the noise of the TAC induces variability in *ϕ*_*1*_ and *ϕ*_*2*_ but not in *θ*. Increasing *n* corrects this problem. For very low noise (*sf = 1*), when *n*<500, only two BFs with neighbor exponents are chosen and the distributions of rate constants presents more than one peak.

### Probability distribution of *K*_i_ as a function of *sf* and [*θ*_min,_ *θ*_max_]

**Fig 2:**
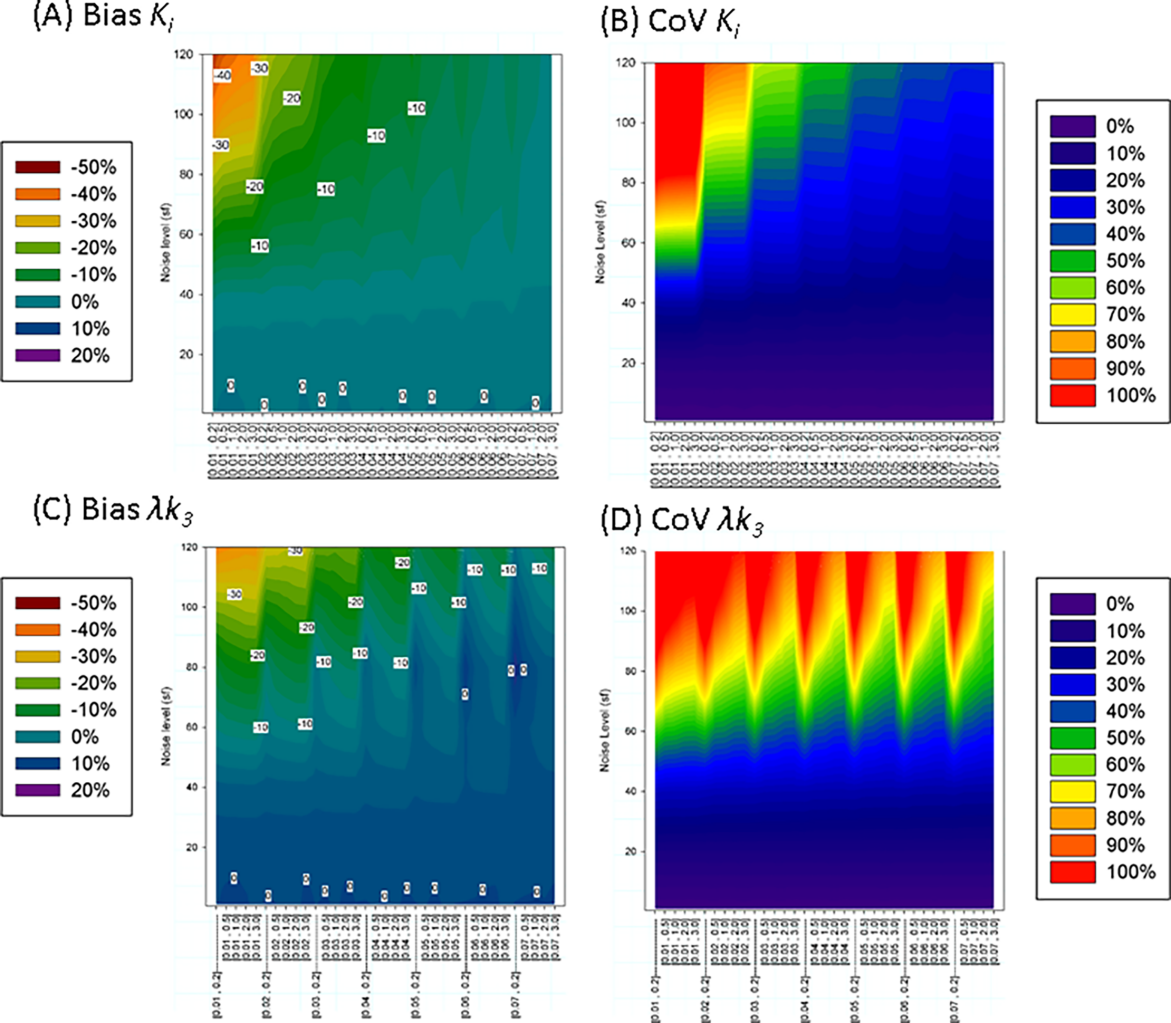
Visual representation of the simulated results using BAFPIC with Vb≡5%. Each vertical line represent the effect of noise in the bias and coefficient of variation (CoV) for a given range [*θ*_min,_ *θ*_max_] of the basis functions. Noise is expressed as the value of the scale factor in Eq 5. Results show that bias and CoV depend more on *θ*_min_ than *θ*_max_.

A visual presentation of the bias and CoV of *K*_i_ and λ*k*_*3*_ is presented in Fig 2. For *sf* = 20, *K*_i_ showed a non-biased normal distribution (bias<1%,*CoV*~6%, *skew* ~-0.3, *kurt*~3.1–3.5) practically independent of *θ*_min_ and *θ*_max_.

At higher levels of noise, the variability of *K*_*i*_ progressively increases and an underestimation of *K*_i_ progressively appears. The distribution of probability of *K*_i_, becomes a non Gaussian shape (negative *skew and kurt*>3). This effect depends more strongly on *θ*_min_ than on *θ*_max_ (i.e more underestimation when slower frequencies of the BF are included, but the faster frequencies play a secondary role). Reasonable normal distributions (-1<*skew*<0, 3<kurt< = 4) can be seen for basis function ranges [0.04 < = *θ*_min_< = 0.07, *θ*_max_> = 0.2] min^-1^. At these ranges, for a given noise level, bias and *CoV* increase nearly linearly when *θ*_min_ decreases and it is practically independent of *θ*_max_ (Fig 2A). [*θ*_min_≥0.06, *θ*_max_ ≥1] minimized the underestimation and *CoV* (e.g. for [0.06, 3] min^-1^ the bias is limited to -5.6% and CoV = 40% for the maximum noise studied (*sf* = 120)). On the other extreme, the worse scenario is for *θ*_min_ ≤0.02 min^-1^ (e.g. for [0.02, 3] min^-1^ the underestimation was quadruple and *CoV* double compared to [0.06, 3] min^-1^).

### Probability distribution of λk_3_ as a function of *sf* and [*θ*_min,_ *θ*_max_]

At *sf* = 20, the noise level of a TAC of a very small ROI, *λk*_*3*_ shows a normal distribution without bias and *CoV*~10% at any BF range.

For *sf*≥40, *λk*_*3*_ distributions showed a high kurtosis (e.g. 4≤*kurt*≤ 8 for *sf* = 40, 8≤*kurt*≤ 145 for *sf* = 60) and for *sf*>60 the distribution showed a high skewness as well.

**Fig 3:**
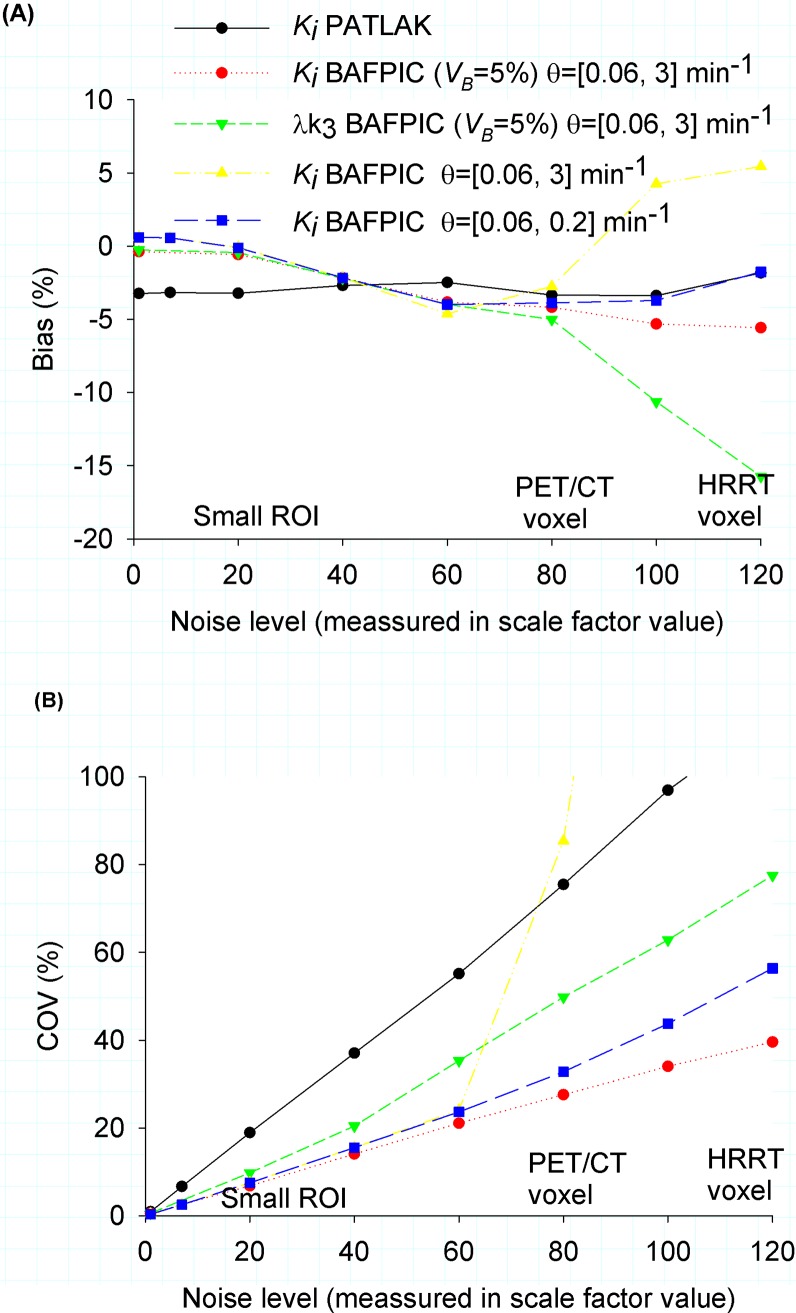
Bias = (mean(*K*_i_ simulation)/*K*_i_ simulated-1)% and *CoV* = std(*K*_i_ simulations)/mean(*K*_i_ simulation) as a function of the noise level (Eq 5). As a reference, we included the approximate voxel noise level of a PET/CT camera (Biograph HiRez XVI. Siemens Molecular Imaging) and a HRRT (CPS/Siemens, Knoxville, TN, USA).

Similar to the case of *K*_i_, *λk*_*3*_ bias depends more strongly on the selection of *θ*_min_ than *θ*_max_. Bias (underestimation) can be reduced by selecting the range of *θ* (higher *θ*_min_ and lower *θ*_max_); however, the COV follows the opposite trend (e.g at higher noise (*sf* = 120) for *θ =* [0.06, 3] min^-1^, the bias = -15% and *CoV =* 77% and for *θ* = [0.06, 0.2] min^-1^, bias = -12% and CoV = 103%). It should be noted that, at the same noise level, even a “low” *CoV* for *λk*_*3*_ is higher than a typical *CoV* for *K*_i_ (Fig 3, Fig 2B vs Fig 2D).

### Effects of *V*_*B*_ fixed to a wrong value on the probability distribution of *K*_i_

In the previous section, we fixed *V*_*B*_ = 5% and $V_{B}^{TAC}$ = 5%. In the present section, *K*_i_ distributions were analyzed when TACs were simulated with $V_{B}^{TAC}$ = 0%, 5%, 10% and 25% and BAFPIC was implemented with *V_B_* = 5%. Simulations were done at high noise levels (*sf* = 100 and 120) and BAFPIC using [0.06, 3] min^-1^.

The result (S1 File, sheet “VB”) demonstrated that an error of X = $V_{B}^{TAC}-V_{B}$ percentage points in *V*_*B*_ will induce an extra bias in *K*_i_ of approximately -X%. Interestingly, for $V_{B}^{TAC}$ = 0, (X = -5), the noise-induced underestimation will cancel out the overestimation due to the error in the *V*_*B*_.

### Probability distribution of *K*_i_ and *λk*_*3*_ for different Bmax

In this section, BAFPIC with *V*_*B*_ = 5% and *θ* = [0.06, 3] min^-1^ and *θ* = [0.07, 3] min^-1^ was used on simulated highly noisy TACs (*sf* = 120). *k*_*3*_ was changed to model different FAAH activities; $k_{3}/k_{3}^{P}$ = 0.1, 0.2, 0.35, 0.5, 0.75, 1, 1.25, 1.5 and 2 were studied. At higher values of *k*_3_, the irreversible radioligand start to show delivery limitation effects.

**Fig 4:**
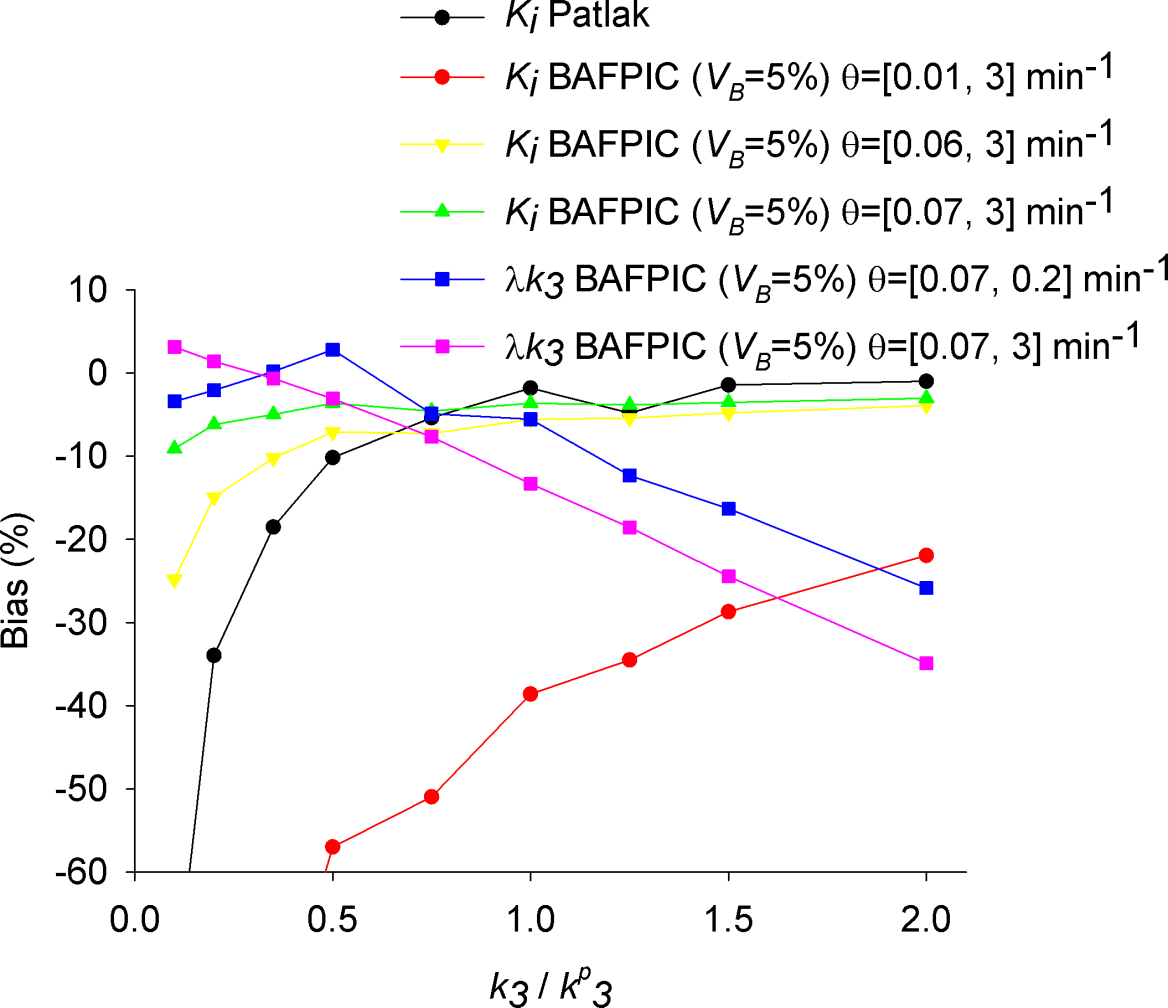
Noise induced bias for *K*_i_ and *λk*_3_ estimated by BAFPIC and for *K*_i_ estimated by Patlak plot as function of $k_{3}/k_{3}^{P}$. The simulated noise corresponds to TACs that are regularly observed at the HRRT voxel level (*sf* = 120). Bias is computed as ((measured-simulated)/simulated)%. Note that the yellow line in this figure corresponds to the yellow line in Fig 5.

Results showed that *K*_i_ presents a relative bias that depends on the value of *K*_i_ and the BF range (Fig 4, green and yellow lines). For *θ* = [0.06, 3] min^-1^, when *k*_*3*_ is in the range 0.35<$k_{3}/k_{3}^{P}$< 2, *K*_i_ bias changes from ~-10% to ~-4%. But when $k_{3}/k_{3}^{P}$< 0.35, corresponding to the cases when the noisy TAC can be fitted equally well by a 1TCM and 2TCMi, the *K*_i_ bias and *CoV* become more pronounced (Fig 4 yellow line). In contrast for *θ* = [0.07, 3] min^-1^, the underestimation of *K*_i_ is lessened and bound within 10% for any *k*_*3*_ (Fig 4, green line). It should be noted that the use of relative bias (%) and CoV can be misleading for small *k*_*3*_ values, and actually the absolute bias and standard deviation present the opposite trend (S1, S2 and S3 Figs).

*λk*_*3*_ presented a more complex and varied pattern of bias under *k*_*3*_ changes and the *θ* range used (Fig 4, blue and purple lines). Importantly, the bias is bound within ±5% for small changes around $k_{3}^{P}$ (e.g. *λk*_*3*_ bias increases from ~-8% to ~-19% when $k_{3}/k_{3}^{P}$ increases from 0.75 to 1.25 using *θ* = [0.07, 3] min^-1^ and the direction of change is such that a simulated reduction of ~40% would be measured by BAFPIC as attenuated to ~32%). CoV(*λk*_*3*_) presented a minimum >67% for $k_{3}/k_{3}^{P}$~0.5 (S1 Fig).

### Probability distribution of *K*_i_ for low and high rCBF and *V*_*ND*_ values as a function of *B*_*max*_

**Fig 5:**
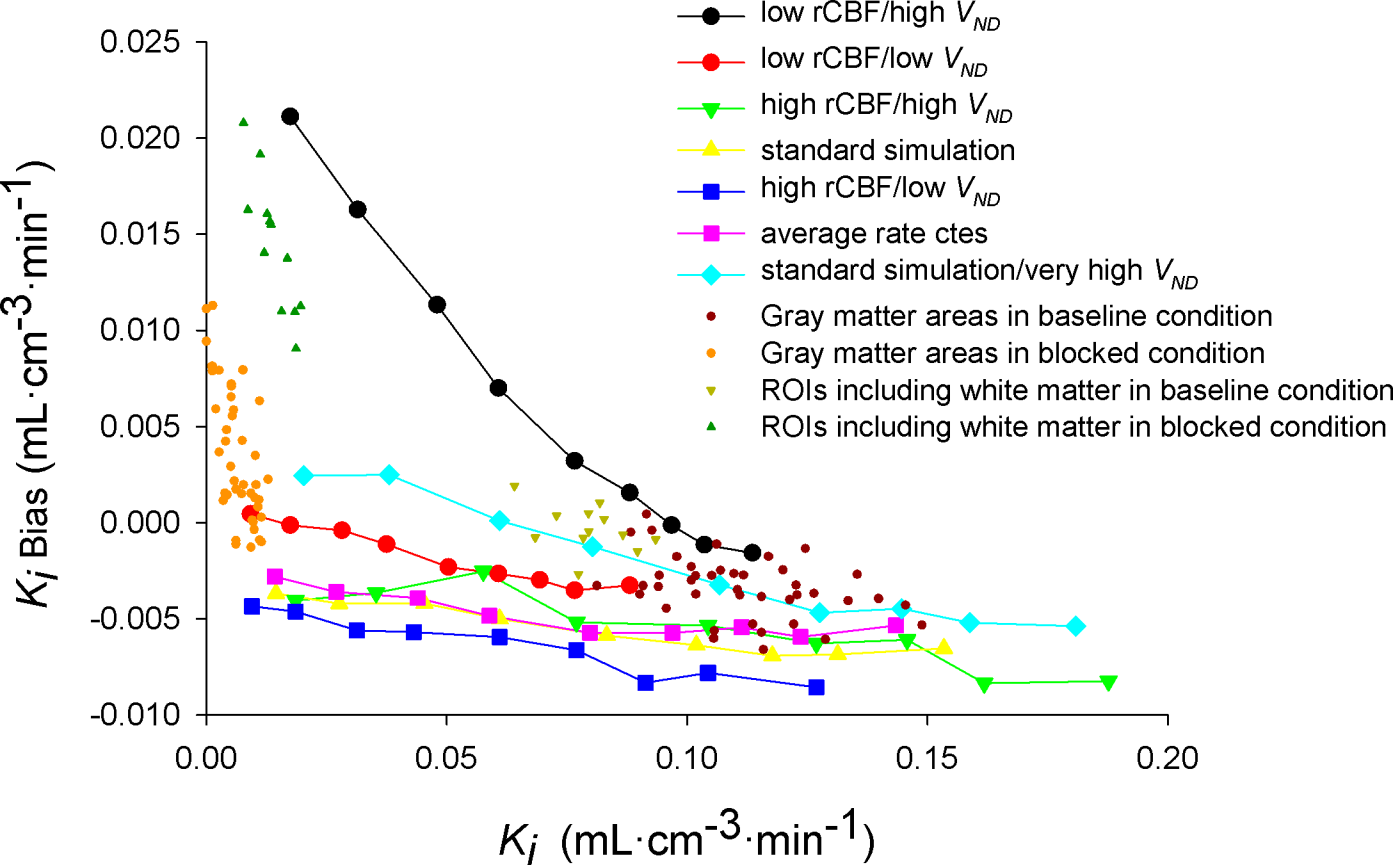
Bias as a function of *K*_*i*_. The simulated results are represented by the solid symbols connected with lines. In these cases, the x-axis represents the simulated *K*_*i*_ value while the y-axis is the mean *K*_*i*_ of the simulation minus the simulated value. Symbols along the line are simulations with the same *K*_*1*_ and *V*_*ND*_ but a different *k*_*3*_. *K*_1_ = 0.16 mL·cm^-3^·min^-1^ (Low rCBF), *K*_1_ = 0.36 mL·cm^-3^·min^-1^ (high rCBF), *V*_*ND*_ = 2, 4 and 4.5 mL·cm^-3^(Low, High, very high respectively). Scattered points are data from real images (6 subjects/9 ROIs/2 scans per subject). In these cases, *K*_*i*_ in the x-axis is the result of the 2TCMi on the regional TAC while the y-axis is the regional mean of *K*_*i*_ in the BAFPIC based parametric map minus the regional 2TCMi estimation. BAFPIC was applied with 50 function with θ in the range [0.06 3] min^-1^ and *V*_*B*_ = 5%.

These simulations were only performed for *V*_*B*_ = 5%, $V_{B}^{TAC}$ = 5%, noise level *sf* = 120, and BF with *θ* = [0.06, 3] min^-1^. The distribution shape of *K*_i_ is reasonably Gaussian, based on kurtosis and skewness. A systematic bias of *K*_i_ as a function of a simulated *K*_i_ value is observed in the continuous lines of Fig 5. At high *k*_3_ values, all the simulations underestimated *K*_i_. At a low *k*_3_, the underestimation of *K*_i_ was lower (Fig 5, blue, green and red lines) or in some cases there was an overestimation (Fig 5, cyan and black lines). Bias values depend on the simulated rCBF and *V*_*ND*_. While for most of the simulations the bias was limited to *circa* ±5% of the baseline $K_{i\left(k_{3}^{}/k_{3}^{P}=1\right)}$, a particularly marked overestimation was observed for the case of low rCBF/High *V*_*ND*_ (Fig 5, black line, *K*_*1*_ = 0.16 mL/cm^3^/min, *k*_2_ = 0.04 min^-1^) when *k*_3_ is low as a consequence of *θ*_min_ = 0.06 min^-1^ > *k*_2_*+k*_3_.

### Simulation results using BAFPIC with *V*_*B*_ variable

**Fig 6:**
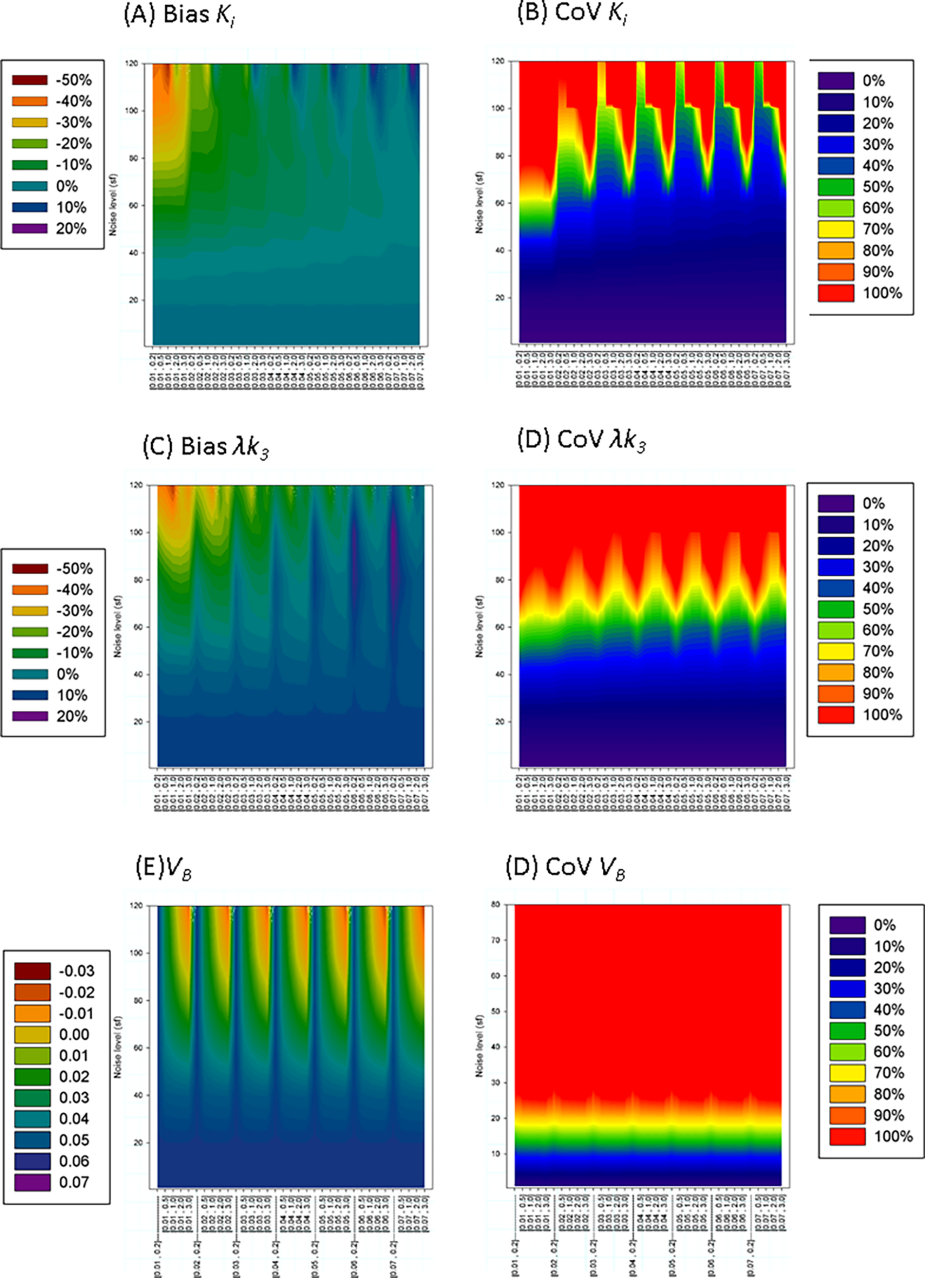
Visual representation of the simulated results using BAFPIC with variable *V*_*B*_. Each vertical line represent the effect of noise for a given range [*θ*_min,_ *θ*_max_] of the basis function. Noise is expressed as the value of the scale factor in Eq 5. Results in A to D show that bias and CoV of *K*_*i*_ and *λk*_*3*_ depend more on *θ*_min_ than *θ*_max_. In contrast plot E shows that at high noise levels, *V*_*B*_ is practically determined by the “high frequency” *θ*_max_ (simulated $V_{B}^{TAC}$ = 0.05).

Fig 6 represents the summarized result of the simulations.

### Probability distribution of *V*_*B*_ as a function of *sf* and [*θ*_min,_ *θ*_max_]

Members of the basis function with higher frequencies look more similarto the input function (Fig 1). High frequencies are related to the rapid transit time of the tracer in the vasculature within the ROI and effects of dispersion in the arterial line [13]. In presence of noise (sf>40), the mean value of *V*_*B*_ is practically determined by *θ*_max_ (Fig 6E) and *θ*_max_ between 0.2 min^-1^ and 0.5 min^-1^ keeps *V*_*B*_ closer to 5% (minimized the bias). Independent of the range of the basis, *V*_*B*_ presents a Gaussian distribution with high variability (for the optimal *θ* range for each sf, *CoV* increases from ~140% at *sf* = 40 to *CoV*~400% at *sf* = 120) (Fig 6D).

### Probability distribution of *K*_i_ as a function of *sf* and [*θ*_min,_ *θ*_max_]

At noise levels of a typical ROI (*sf* = 7) or even of a tiny ROI (*sf* = 20), *K*_i_ shows a normal distribution with low bias (<1%) and *CoV* = 2.5% and 7.5 for *sf* = 7 and *sf* = 20, respectively (Fig 6A and 6B, Fig 3A yellow and blue lines). For higher noise levels (*sf*≥40), the shape of the distribution (kurtosis and skewness) depends strongly on the range of *θ* considered. The distributions are leptokurtic (*kurt* >3). The kurtosis goes to 3 for higher *θ*_min_. The bias of the mean is always negative (underestimation) and becomes stronger for higher *θ*_max_. The *CoV* (and skewness) presents a minimum in the range 0.2< *θ*_max_ <1 for each *sf* and *θ*_min_. *θ* = [0.06, 0.2] min^-1^ presents a good trade off, keeping a distribution shape closer to the normal and reducing bias and *CoV* (eg. for *sf* = 100, bias = -3.7%, *CoV* = 43%, kurt = 7, *skew* = 1.1)

While *CoV*(*K*_i_) increases linearly with the noise level when applying BAFPIC with a fixed *V*_*B*_ (Fig 3B, red line), it increases exponentially when *V*_*B*_ is variable, using the optimal BF sets for each case (Fig 3B, blue line). For *sf*< = 60, the difference is not important, but for *sf* = 120, *CoV*(*K*_i_) ≈40% using *V*_*B*_ constant vs *CoV*(*K*_i_) ≈56% using *V*_*B*_ variable.

### Probability distribution of *λk*_*3*_ as a function of *sf* and [*θ*_min,_ *θ*_max_]

BAFPIC estimations for *λk*_*3*_ when *V*_*B*_ is variable were similar to those when *V*_*B*_ is constant, but with higher *CoV*s (Fig 6C and 6D). At *sf* = 20, *λk*_*3*_ shows a normal distribution without bias and *CoV*~11% for any base function considered. For *sf*≥40, *λk*_*3*_ distributions showed a high kurtosis (8≤*kurt*≤11 for *sf* = 40, 17≤*kurt*≤ 300 for *sf* = 60 and higher for higher noise). For *sf*≥60, the distribution presents a high skewness as well. For sf>60, the variability is so large (*CoV*>67%) that the precise bias (usually underestimation) is difficult to determine. The bias depends more strongly on the selection of *θ*_min_ than *θ*_max_; higher *θ*_min_ decreases the underestimation. For each *θ*_min_, *CoV* is minimized for a *θ*_max_ between 0.5≤ *θ*_max_ ≤ 2 min^-1^ (e.g. for *sf* = 120 and *θ* = [0.06, 0.5], min^-1^ the bias is -11% and CoV = 126%.

### Patlak plot as a function of *sf* and *B*_*max*_

Patlak plot reached linearity for t* = 27.25 min in TACs with $k_{3}/k_{3}^{P}$ = 1. Using this t*, $K_{i}^{Patlak}$ showed a low underestimation (between -2% and -5%), practically independent of *sf*. The *CoV* of $K_{i}^{Patlak}$ increased linearly with *sf*. *CoV* was more than 3 times higher than *CoV* of *K*_i_ with BAFPIC using a constant *V*_*B*_ (Fig 4, black vs red line). For *sf* = 120, the mean of $K_{i}^{Patlak}$ was still fluctuating within ±2% after 5000 simulations.

At *sf* = 120, changes in *k*_3_ did not induce a significant effect on the bias in the range of 0.5≤ $k_{3}/k_{3}^{P}$≤ 2 (Fig 4, black line). However, for $k_{3}/k_{3}^{P}$< 0.5, when the TAC can be fitted with 1TCM, a significant underestimation appeared reaching -76% for $k_{3}/k_{3}^{P}$ = 0.1.

### BAFPIC with constant *V*_*B*_ = 5%: Application to real images

**Fig 7:**
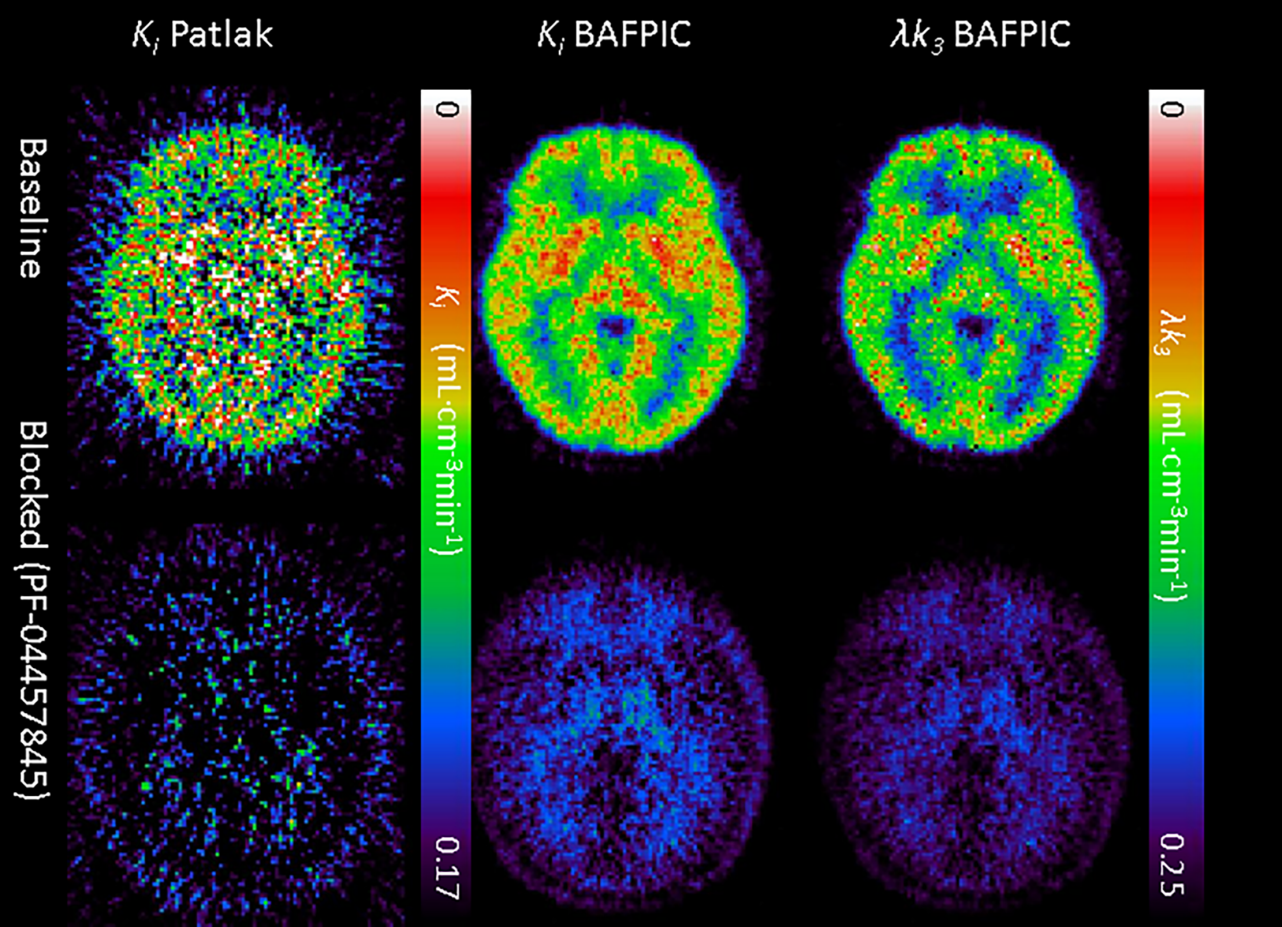
Axial slice (MNI z = +2mm) of the averaged (n = 6) parametric maps of *K*_*i*_ (Patlak and BAFPIC) and *λk*_*3*_ (BAFPIC). The images in the upper row are at baseline condition, while the images on the lower row were acquired 2 hours after an oral dose ≥1 mg of PF-04457845. Note that while the gray matter changed substantially, the change is less marked in the white matter. Units are mL·cm^-3^·min^-1^.

Parametric maps were generated for 6 subjects in the baseline and block conditions using the optimal parameters found in the simulations (*θ =* [0.06, 3] min^-1^, *#basis functions = 50*, Fig 7). Histograms of values inside the large homogeneous ROIs (e.g. cerebellum cortex, ~10^4^ voxels) showed normal distributions (S4 Fig). Mean values in the parametric maps of each ROI correlated excellently with estimations given by the ROI analysis (r^2^ = 0.992 including all ROIs in baseline and blocked conditions, and r^2^ = 0.996 in the baseline condition only). Simulation-predicted bias as a function of *K*_*i*_ value was observed (Fig 5, color symbols). Overall the bias in Fig 5, including all ROIs data points, can be fitted by the linear regression: $\langle K_{i}^{par}\rangle -K_{i}^{ROI}=-0.092\;K_{i}^{ROI}+0.0072\;\min^{-1}$ mL·cm^-3^·min^-1^ for 0<$K_{i}^{ROI}$<0.15 mL·cm^-3^·min^-1^. However, this relationship is more complex than linear; in a blocked condition, regions containing white matter (Pons and Middle brain) showed a higher overestimation using BAFPIC than ROI, most likely due to *k*_2_*+k*_3_<0.06 min^-1^. On the other hand, it should be noted that in the blocked condition, the gray matter regions present a large dispersion in the bias without a clear pattern. The bias slightly decreases the differences measured in the parametric map (e.g. for a ROI with a $K_{i}^{ROI}$ of 0.1 a change ±20% would be measured in the parametric maps as ±18.5%, a change of ±40% as ±37%, and a change of ±90% as ±83%). However, for a WM area, the linear relationship is not maintained (see Fig 5): a reduction of 83% from $K_{i}^{ROI}$ = 0.06 to 0.01 would be measured in the parametric ~60% rather than the linear prediction of 73%. In summary, the bias would strongly depend on the relation of *k*_*2*_*+k*_*3*_with *θ*_min_.

The bias found in the real data follows approximately the pattern predicted with the simulations. WM regions (low rCBF) fall in the middle of the simulations of low rCBF/high V_ND_ and low rCBF/low V_ND_. However, gray matter ROIs behave as having a V_ND_ = 4.5 mL/mL, which is higher than reported in Rusjan *et al*. [1], yet closer to what was measured in blocking conditions in Boileau *et al* [2].

Previously, we have published in our gray matter ROI analysis a reduction of λ*k*_3_>90% after 2 hours of an oral dose of ≥1 mg of PF-04457845[2]. It should be noted that while λ*k*_3_ is proportional to *k*_3_, *K*_i_ is not directly proportional and is less sensitive to changes in *k*_3_ depending on the ratio of *k*_3_/*k*_2_. A 90% reduction in *k*_3_ should reduce *K*_i_ by 82% when *k*_3_ = *k*_2_ (e.g. gray matter) and 75% when *k*_3_ = 2*k*_2_ (e.g. white matter).

**Fig 8:**
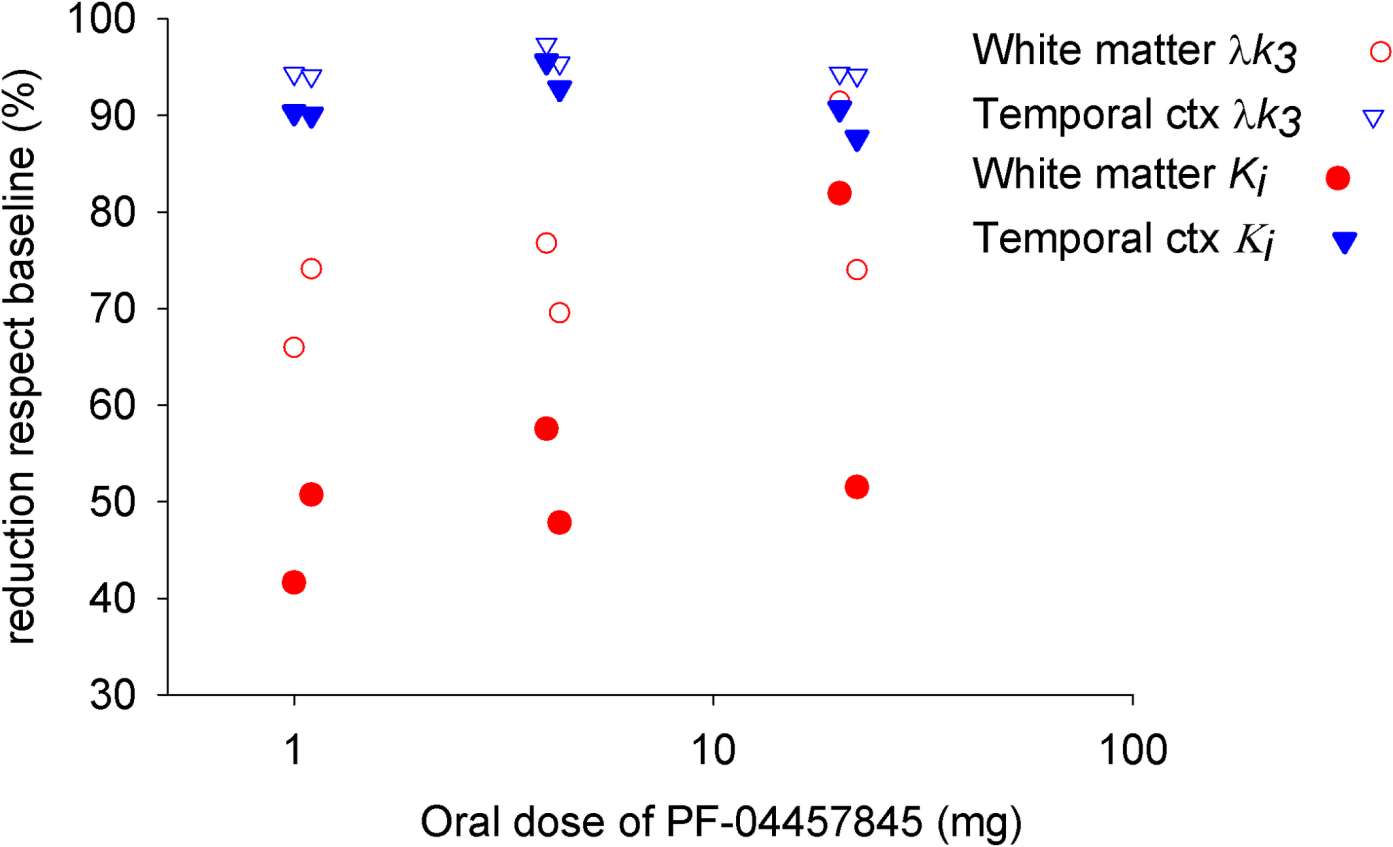
The change in FAAH activity, as measured with λ*k*_3_ (open symbols), following an oral dose of 1, 4 and 20 mg of PF-04457845 in the gray matter (temporal cortex) and white matter of 6 subjects. Solid symbols show the relative reduction of [^11^C]CURB *K*_*i*_ in the same experiments. A small offset was applied for each oral dose in order to visualize overlapping symbols.

Fig 7 represents the average parametric *K*_*i*_ maps of the 6 subjects in the baseline and blocking conditions. In the gray matter, the reduction of *K*_i_ in the blocking condition is above 80%, which is consistent with our previously published result of a reduction of λ*k*_3_>90%. In contrast, the reduction in WM is merely ~40%, from $\langle K_{i}^{baseline}\rangle =0.057$ mL·cm^-3^·min^-1^ to $\langle K_{i}^{block}\rangle =0.034$ mL·cm^-3^·min^-1^. These *K*_i_ values are within the range in which the bias behaves linearly and correspond to a reduction in *k*_3_ of 45%-50%. In order to confirm these results, a large ROI of the WM was delineated (see methods) and the TAC was quantified using 2TCMi (Fig 8). While occupancy in gray matter was > 95% and was practically dose-independent, the white matter presented a lower occupancy that was apparently dose dependent. Fig 7 shows that parametric maps of *K*_*i*_ estimated with Patlak or with λ*k*_3_ estimated with BAFPIC are more pixelated than *K*_*i*_ estimated by BAFPIC as a consequence of higher variability.

### BAFPIC with variable *V*_*B*_: Application to real images

Parametric maps using BAFPIC, with variable *V*_*B*_, were consistent with the results of the simulations. BF with *θ* = [0.06, 0.2] min^-1^ provides realistic *V*_*B*_ values (~5%) with high variability. In contrast, the average parametric *K*_i_ values correlate weaker with the ROI estimation than when BAFPIC with a fixed *V*_*B*_ is used. Correlation of *K*_i_ becomes stronger for the BF with *θ* = [0.06, 0.5] min^-1^, but estimations of *V*_*B*_ becomes too low to be considered realistic. *V*_*B*_ in the block condition is lower and more variable than in baseline. It should be remarked that *V*_*B*_ loses identifiability in the ROI analysis for the blocked TAC as well.

## Discussion

The present work examined the bias induced by the noise when using BAFPIC to quantify voxel wise images of [^11^C]CURB, a 2TCM irreversible radioligand. This work explicitly demonstrated the validation and limitations of the parametric maps of [^11^C]CURB as a function of the range [*θ*_min,_ *θ*_max_] when brain images are acquired with an HRRT and reconstructed using a FBP algorithm with Hann filter at Nyquist frequency.

The high level of noise in HRRT images does not allow for much flexibility in the selection of the member of the basis functions. The selection of [*θ*_min,_ *θ*_max_] affects the shape of the distribution of probability of the parameters of interest (*K*_i_ and λ*k*_3_). Noise induces a systematic bias in the mean of the distribution of *K*_i_ and λ*k*_3_, which would underestimate differences in FAAH concentration. From a parametric mapping perspective, it is convenient to focus on *K*_i_ rather than λ*k*_3_. Variability of *K*_i_ is lower and the dependence of the bias with a change in *B*_*max*_ is lower. BAFPIC strongly reduces the variability of the standard method (Patlak plot).

For parametric maps, it is convenient to apply BAFPIC with *V*_*B*_ fixed rather than variable. At those high levels of noise, *V*_*B*_ does not present identifiability (*CoV*~400%) its mean values are a function of the highest frequency in the basis (*θ*_max_, c.f. section “high frequency components” in ref [13]) and the macroparameters derived when *V*_*B*_ is variable present higher variability. While a fixed *V*_*B*_ (e.g. to 5%) is incorrect in a voxel corresponding to a big artery (e.g. *V*_*B*_ = 100%), techniques of analysis of parametric images look for cluster of voxels rather than single voxels, thus this error is nearly negligible. It should be noted that in the graphical methods for parametric mapping (i.e. Patlak or Logan plot), it is common practice to either ignore or subtract the vascular contribution with a fixed *V*_*B*_ value from the TAC prior to the application of the model.

An exaggerated selection for the slowest frequencies (e.g. *θ*_*min*_ = 0.01 min^-1^) will produce non-normal distributions of *K*_i_ and λ*k*_3_ with a mean presenting significant underestimations. Our results are consistent with previous work that has shown: 1) that the spectral analysis method is known to be affected by the cutoff for slow frequencies[15] and 2) that using SRTM (BFM), the non-normal distribution of rate constants using BF can be observed for $k_{2}^{\prime }$ [25].

Based on the simulations, we found that the parametric images of *K*_i_ can be generated with BAFPIC using constant *V*_*B*_ (= 5%) and a BF of *n*≥50 members with *frequencies* distributed logarithmically within [0.06, 3] min^-1^. Consistently, our real images demonstrated that these settings were useful to look at 90% changes in the gray matter. Despite having a lower ability to quantify similar changes in the white matter, the potential of the parametric maps analysis was illustrated after the revelation of results in the white matter following FAAH inhibition with PF-04457845 that were not hypothesized in the ROI analysis. In clinical populations, in which changes in *B*_max_ are expected to be moderate (e.g. subjects with single nucleotide polymorphism (rs324420, C385A) show 23% lower [^11^C]CURB binding (*λk*_3_) in brain[26]), it is expected that those settings for BAFPIC will work well for both gray and white matter ROIs. However, the results presented in this work will eventually allow to customize the BAFPIC parameters for different scenarios (macroparameters, expected values of rate constants based on rCBF, *V*_*ND*_ and *B*_max_), while understanding the limitations.

The mathematical expression in the 2TCMi of λ*k*_3_ has advantages over *K*_i_ a) it is proportional to *k*_3_ (B_max_), and b) it does not depend explicitly on rCBF. Despite the fact that all the irreversible radiotracers can suffer from delivery limitation effects, the proportion between rate constant (*k*_3_~*k*_2_/2) for healthy controls is such that in the gray matter, [^11^C]CURB binding is not sensitive to rCBF. The white matter (*k*_3_~*k*_2_) might be more compromised; however, the TACs still present a peak followed by a plateau that allows to differentiate the contribution of the delivery and specific binding. In contrast, *K*_i_ is, by definition, affected by rCBF. In [^11^C]CURB, its effect is not strong: a significant change of rCBF from 90 mL·100 mL^-1^·min^-1^ (*K*_1_ = 0.28 mL·cm^-3^·min^-1^) to 40 mL·100 mL^-1^·min^-1^ (*K*_1_ = 0.22 mL·cm^-3^·min^-1^)[1] would reduce *K*_i_ by 10% (using a high *V*_*ND*_ = 4 ml·cm^-3^ and $k_{3}^{p}$). When expected changes in rCBF may affect the interpretation of the results of a study, parametric maps of λ*k*_3_ are still feasible but the optimal range for the BF is [0.06, 0.5] min^-1^. However, the statistical power will decrease as a consequence of the increase in variability and bias correlated with *k*_3_ values.

There is a discrepancy between the simulation and the real data regarding *V*_*ND*_. Fig 5 seems to indicate that the real data in the baseline condition (*V*_*ND*_~3 mL·cm^-3^) corresponds to the simulated data with *V*_*ND*_ = 4.5 mL·cm^-3^. We have also observed in the ROI analysis that 1) *V*_*ND*_ in the blocked condition is slightly higher[2], 2) with >90% specific binding blocked 2TCMi still fits the TACs better than 1TCM, 3) 1TCM model of the blocked TAC gives a slightly higher V_ND_. Therefore, the simulated TACs with 2TCMi (1 reversible compartment for non displaceable binding and other irreversible for specific binding) could be an oversimplification. On the other hand, a straightforward comparison may be affected by a number of factors: 1) simulated changes in V_ND_ kept *k*_3_ fixed and simulated changes in *B*_max_ kept *k*_2_ fixed, but in our experiments we noted that *k*_2_ correlated with *k*_3_ in healthy controls, 2) while data points in the simulation were weighted based on Eq 5, in the real data true concentration C_i_ is unknown, thus the weight is based on trues in the field of view, 3) simulated noise level could overestimate/underestimate the noise level of the HRRT, 4) tissue heterogeneity in the ROI could affect the comparison between parametric maps and ROI results.

The results presented here for TACs with intermediate noise level can be applied to [^11^C]CURB images acquired with different scanners or algorithms for reconstruction. However, it should be noted that the simulations presented here assumed Gaussian noise, which eventually may not be the case for images coming from other algorithms of reconstruction (e.g. non-negative constrained iterative algorithm when the number of counts in detectors is low).

The present results can provide a guideline for application to other radioligands; however, every radioligand will require an independent validation. For example, for a radioligand with a slower delivery to tissue, the vascular component may be more visible in the TAC and *V*_*B*_ can be fitted.

## Conclusion

BAFPIC with constant *V*_*B*_ can be used to generate *K*_i_ parametric maps of [^11^C]CURB images acquired with the HRRT provided that the range of the BF is carefully adjusted. The noise induces an underestimation proportional to the FAAH concentration, which will reduce the potential differences between groups. Thus, BAFPIC reduces more than 60% the variability relative to the Patlak plot. While λ*k*_3_ parametric maps are feasible, they present higher bias and variability. In images with lower noise (e.g. different scanner or reconstruction algorithm) these effects will decrease.

## Supporting information

S1 FigNoise induced variability expressed as CoV = 100%*Stdev/mean for *K*_i_ and λ*k*_3_ as a function of $k_{3}^{}/k_{3}^{P}$ for BAFPIC with different ranges of the exponent *θ* of the basis function compared to Patlak plot.The simulated noise in the TACs is similar to the noise regularly observed at the HRRT voxel level (*sf* = 120).(TIF)

S2 FigNoise induced variability expressed as Stdev for *K*_i_ and λ*k*_3_ as function of $k_{3}^{}/k_{3}^{P}$ for BAFPIC with different ranges of the exponent *θ* of the basis function compared to Patlak plot.The simulated noise in the TACs is similar to the noise regularly observed at the HRRT voxel level (*sf* = 120).(TIF)

S3 FigNoise induced bias for *K*_i_ and λ*k*_3_ as function of $k_{3}^{}/k_{3}^{P}$ for BAFPIC with different ranges of the exponent *θ* of the basis function compared to Patlak plot.The simulated noise in the TACs is similar to the noise regularly observed at the HRRT voxel level (*sf* = 120). Bias is computed as (measured-simulated). c.f. Fig 3 in manuscript. Note that yellow line in this figure corresponds with yellow line in Fig 4 in manuscript.(TIF)

S4 Fig Histograms of *K*_i_ values in the cerebellar cortex of a single subject parametric map. Comparison of the distribution produced by BAFPIC (*V*_*B*_ = 5%) *θ =* [0.06, 3] min^-1^, BAFPIC (*V*_*B*_ = 5%) *θ =* [0.01, 3] min^-1^, and Patlak plot. The mean values are indicated with triangles together with the *K*_i_ estimation from 2TCMi for the ROI analysis. While the Patlak distribution presents no bias, it shows a higher variability. The skewed distribution for BAFPIC (*V*_*B*_ = 5%), *θ =* [0.01, 3] min^-1^ produce a large bias in the mean values of the distribution. BAFPIC (*V*_*B*_ = 5%), *θ =* [0.06, 3] min^-1^ gives a tradeoff between bias and variability.
(TIF)

S1 File Monte Carlo simulations. This MS-Excel file contains all the simulations performed.
(XLSX)
