# Supplementary figures and images for: Voxel level quantification of [11C]CURB, a radioligand for Fatty Acid Amide Hydrolase, using high resolution positron emission tomography
Source: PLoS One. 2018 Feb 14;13(2):e0192410. doi: 10.1371/journal.pone.0192410 (PMC5812639; doi:10.1371/journal.pone.0192410)

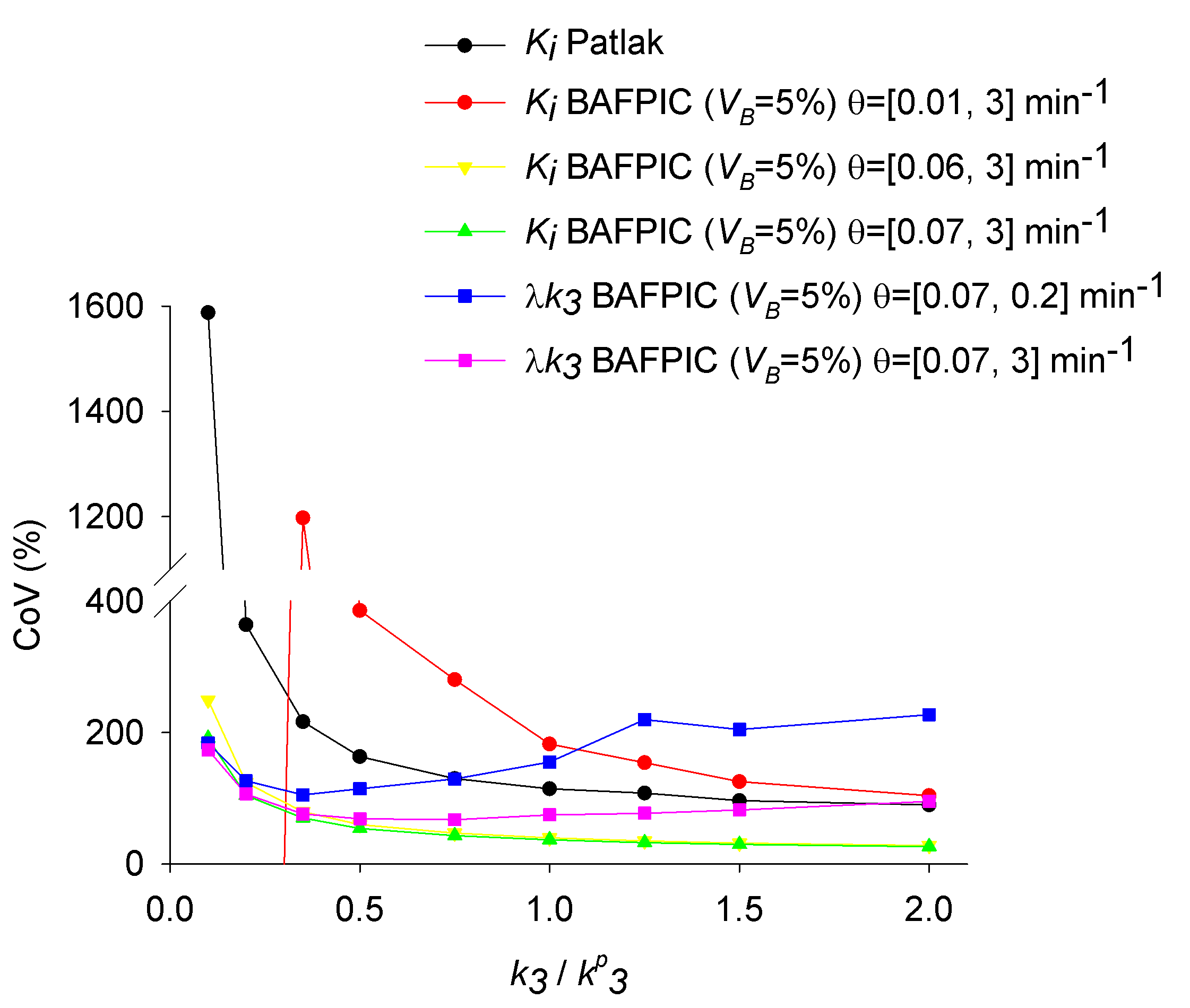

Supplement: S1 Fig — The simulated noise in the TACs is similar to the noise regularly observed at the HRRT voxel level (sf = 120). (TIF) [file pone.0192410.s001.TIF]

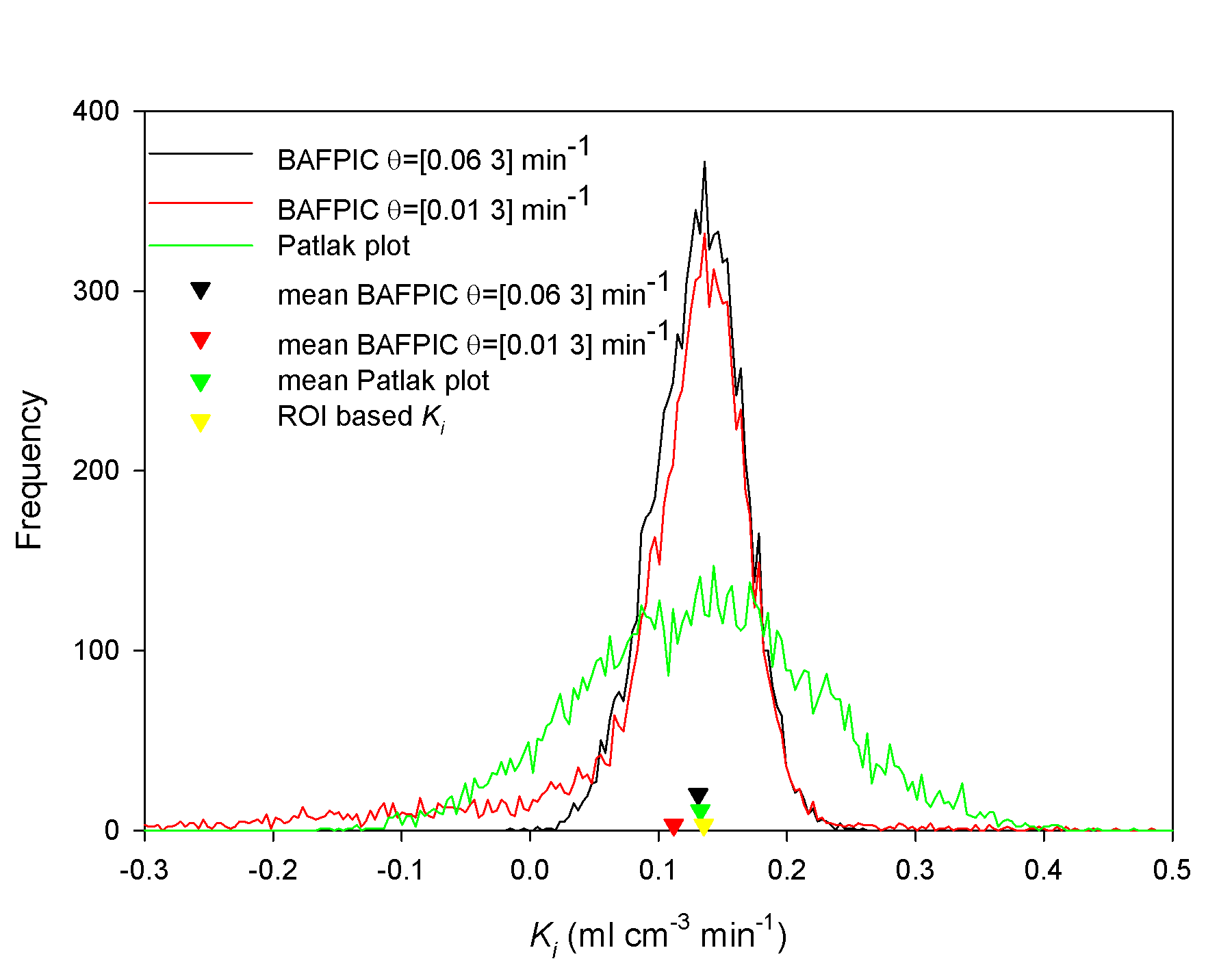

Supplement: S4 Fig — Comparison of the distribution produced by BAFPIC (VB = 5%) θ = [0.06, 3] min-1, BAFPIC (VB = 5%) θ = [0.01, 3] min-1, and Patlak plot. The mean values are indicated with triangles together with the Ki estimation from 2TCMi for the ROI analysis. While the Patlak distribution presents no bias, it shows a higher variability. The skewed distribution for BAFPIC (VB = 5%), θ = [0.01, 3] min-1 produce a large bias in the mean values of the distribution. BAFPIC (VB = 5%), θ = [0.06, 3] min-1 gives a tradeoff between bias and variability. (TIF) [file pone.0192410.s004.tif]
